# An Overview of Coverage of BCG Vaccination and Its Determinants Based on Data from the Coverage Survey in Zhejiang Province

**DOI:** 10.3390/ijerph15061155

**Published:** 2018-06-01

**Authors:** Yu Hu, Yaping Chen, Hui Liang, Ying Wang

**Affiliations:** 1 Institute of Immunization and Prevention, Zhejiang Center for Disease Control and Prevention, Hangzhou 310000, China; zjmyscyp@163.com (Y.C.); hliang@cdc.zj.cn (H.L.), ywang@cdc.zj.cn (Y.W.)

**Keywords:** BCG vaccine, coverage, determinants, expanded program on immunization, epidemiology

## Abstract

To assess the Bacille Calmette-Guérin (BCG) vaccination coverage in Zhejiang province and to investigate predictors of the BCG vaccination, we used data from the 2017 Zhejiang provincial coverage survey. Demographic and immunization data on the selected children, their mothers, and their families were also collected by using a pre-tested questionnaire. BCG scars were verified among children who were available at the moment of survey. Coverage of BCG and other expanded program on immunization (EPI) vaccines scheduled before the first year of life was calculated. BCG coverage through the scar assessment and timeliness of BCG were also presented. Multivariate analyses of the predictors associated with the BCG vaccination and its timeliness were conducted separately. In total, 1393 children agreed to participate in the survey and presented the immunization cards. Of them, the coverage of BCG was 92.0% and 88.3% received the BCG within the first 28 days after birth. Besides this, 1282 out of the 1393 children were screened for the BCG scars and 97.1% of them had developed the scars. The multivariable logistic regression analyses indicated that hospital delivery, higher maternal education, a mother with no job, and a resident child were positively associated with the higher BCG vaccination coverage and its timely administrations. BCG coverage was optimal and it was administered in a timely manner. The majority of children vaccinated with BCG developed scars. Tailored interventions should be more greatly focused on and targeted to children with the risk factors identified in this study.

## 1. Introduction

Tuberculosis (TB) remains a global concern of public health as it is one of the leading causes of death by infectious agents worldwide and is responsible for an estimate of over one million deaths annually [1]. Although the efficacy of Bacille Calmette-Guérin (BCG) against pulmonary TB is controversial [2], it is the only available vaccine to prevent the most severe forms of childhood TB, with a vaccine efficacy against TB meningitis and miliary TB of 73% and 77%, respectively [3,4]. A recent study from Norway showed that BCG’s protection was more cost-efficient than expected in a long-lasting situation [5]. Another report from Guinea-Bissau indicated that administering BCG at birth could reduce neonatal mortality by 48% among premature infants [6]. An additional importance of BCG vaccination is its proximity to the delivery and being an entry point to an expanded program on immunization (EPI) and other public health services [7].

The immunization policy of BCG issued by the Chinese center for disease and prevention includes three aspects: first, all newborns need to be administrated BCG at birth, except for a positive or suspicion of HIV infection; second, children three months to three years old without BCG vaccination should be screened by a purified protein derivative (PPD) test, and BCG should be given if the test result is negative; third, children aged ≥4 years without BCG vaccination need not be vaccinated. In China, there is only one licensed BCG vaccine (the D2 strain), which is derived from the strain Glaxo 1077 and manufactured by China Biotech Group Company (Beijing, China).

The development of a scar after BCG vaccination is a useful indicator of immune response [6,8], although there are other impact factors involved in the primary vaccination failure, such as poor cold chain management and inappropriate injection [3]. The widely applied methods for evaluating BCG coverage include the verification of the immunization record [9,10,11,12,13] and direct BCG scar observation [14,15]. Previous studies have indicated that female gender, a lower education level of parents, poor knowledge of immunization and vaccine-preventable diseases, a poor wealth index, and a great number of siblings were predictors of non-vaccination [10,13,15].

China is a high TB burden country with the third highest number of TB cases reported annually worldwide [16,17]. To date, the control strategies of TB include improvement and enhancement of access to medical treatment and prevention through BCG vaccination. The Chinese EPI was launched in 1978 and a complete schedule before 12 months old includes one dose of BCG, three doses of poliovirus vaccine (PV), three doses of diphtheria-pertussis-tetanus vaccine (DPT), three doses of hepatitis B vaccine (Hep B), one dose of measles–rubella combined vaccine (MR), and one dose of Japanese encephalitis virus live attenuated vaccine (JEV). Vaccines included in the Chinese EPI are administered free of charge [18].

Administrative coverage estimates, which are calculated by using the report on vaccinations administered divided by the estimated target population, have remained over 99% in recent years in Zhejiang province. However, the administrative coverage is often unreliable due to incomplete or inaccurate immunization records, duplicate reporting, an outdated population census, and mistakes in compiling monthly summaries of vaccinations [19].

Thus, this study aimed to measure the BCG coverage using the data of the 2017 Zhejiang provincial coverage survey, through BCG records in immunization cards, and by BCG scar observation. Furthermore, we had another three secondary objectives: (1) to analyze the timeliness of BCG to assess whether it was administered in the right time period; (2) to compare the BCG coverage with other vaccine doses schedules before 12 months of age; and (3) to identify risk factors potentially associated with missed or untimely BCG vaccinations.

## 2. Materials and Methods

### 2.1. Study Area

Zhejiang province is on the East coast of China. It covers an area of 101,800 km^2^ and, with a population of 72 million (2016 census), is one of the most densely populated provinces in China. The annual population growth of Zhejiang province is around 10%, with an estimated 726,511 births in 2016. Administratively, it is divided into 11 cities, 90 counties, and 1319 towns.

### 2.2. Target Children

A household-based cluster survey among children aged 24–35 months (born from 1 September 2014 to 31 July 2015) living in Zhejiang province was conducted in August 2017. The main purpose of this coverage survey was to evaluate the coverage of the 17 vaccine doses scheduled before 24 months of age.

### 2.3. Sample Size

The sampling procedure of the 2017 Zhejiang provincial coverage survey was based on the immunization cluster survey recommended by the World Health Organization (WHO) [20]. The formula used to estimate the sample size was as follows:

(1) $$N_{\min}=deff\times \frac{z_{\left(1-\alpha /2\right)}^{2}\times p\times \left(1-p\right)}{d^{2}}$$

According to the survey protocol and to obtain the estimates of full coverage of the 17 doses scheduled before 24 months old at the city level with a two-tailed *α* error of 5%, assuming a precision of 0.1, and the expected full coverage (*p*) at 80%, and a design effect (deff) of 2, the minimum sample size required for each city was 123 eligible children. For the convenience of practical operation, the final sample size was 126 for each city, which was divided into 6 clusters (towns) of 21 children in each cluster. As such, the sample size for the entire province was 1386.

### 2.4. Survey Procedures

First, six towns (clusters) for each city were selected from the list of towns (with the population size of each town) by city on the basis of the probability proportional to population size. Second, one community was randomly selected through a simple ballot from the list of all communities of each selected town. Third, the first household was selected randomly from the list of all households in the selected community by using a table of random numbers. Fourth, we selected the subsequent 20 households by turning to the right while exiting the household and visiting the adjacent households. Only one eligible child per household was randomly selected for the survey. Households were excluded if there were no eligible children or if they appeared vacant. Households in which somebody was living, but without any response, were re-scheduled for another visit. If we could not find 21 eligible children in the selected community, then we moved to the closest community in the same town and repeated the procedures above to survey the remaining children.

### 2.5. Data Collection

A pre-tested questionnaire, which was designed to take less than 15 min, had been developed by the Zhejiang provincial center for disease control and prevention (ZJCDC). Mothers of the selected children were visited at home by trained interviewers. Immunization data were transcribed from immunization cards. Only written records were included in the survey and data analyses. Any child without written evidence of having received BCG vaccination or other EPI vaccinations from an immunization card was considered as not immunized and any child whose parents did not present the immunization card was also considered as not immunized. Demographic information and socio-economic characteristics of the selected children, their mothers, and their families were also collected. In order to assess BCG vaccination coverage through the presence of a scar, we checked the scars of all children enrolled in the survey if they were present at the moment of the interview.

### 2.6. Measurements

Vaccination coverage, of BCG and other EPI vaccines scheduled before 12 months of age, was defined as the proportion of children with a recorded vaccination administered in their immunization cards divided by children whose immunization card was available and assessed. Alternatively, to measure the coverage of BCG through the scar assessment, the vaccination coverage of BCG was calculated through the number of children presenting a BCG scar divided by the total number of children assessed for scarring. Timeliness of BCG was defined as a child receiving BCG before the first 28 days of life [11].

### 2.7. Statistical Analysis

We used STATA 11 (Stata Corp. 2009, Stata statistical software, college station, Lakeway, TX, USA) for data analyses. The description included qualitative variables and quantitative variables categorized according to the objective of the study. The demographic characteristics of surveyed children were described as absolute values and relative frequencies. The vaccination coverage estimates, including those based on verification of the immunization cards and observation of BCG scars, were calculated as a proportion with 95% confidence intervals (CI). The coverage rates of other EPI vaccinations scheduled before one year of age, including the third dose of PV (PV3), the third dose of Hep B (Hep B3), the third dose of DPT (DPT3), the first dose of JEV (JEV1), and MR, were also calculated as the reference.

The cumulative probability of being vaccinated for BCG was estimated at age *t* through the inverse Kaplan–Meier survival function [21], the number of days of delay based on the maximum age at which the vaccination was recommended. Hence, the first day of delay of BCG was defined as the first day after the first 28 days of life. Children who had not received the BCG at the age of 28 days or unvaccinated children were considered as censored. The specific age at which a coverage of 90% was achieved was also evaluated.

Each socio-economic variable which seemed to be potentially associated with the administration of BCG and timeliness of BCG through verification of immunization cards was assessed by a *χ^2^* test with odds ratios (OR) with a 95% CI and *p*-values presented.

A stepwise procedure was carried out in order to build multivariate logistic regression models to evaluate the risk factors of missed BCG vaccination and the timeliness of BCG separately by using those variables with *p*-values <0.1 in the univariate analyses. The final models were fitted using backward selection with a cut-off level at *p* < 0.05.

### 2.8. Ethics Considerations

This study was approved by the ethical review board of ZJCDC (T-037-S). All methods were carried out in accordance with relevant guidelines and regulations. Written informed consent was obtained from every caregiver once there was a decision to participate.

## 3. Results

### 3.1. Demographic of the Surveyed Children

In the 2017 Zhejiang provincial coverage survey, 1511 households with eligible children were visited, and there were 1406 (93.1%) children and their parents who agreed to participate in the survey. Of them, 1393 children presented an immunization card to the field interviewer for transcription of the information on immunization and 1282 children were screened for BCG scars as they were at home during the interview. Of the children whose immunization cards were not available, eleven children declared the specific reasons (e.g., cannot find it/destroyed/lost it), while the other two had never received an immunization card before.

Of the children with immunization cards, 51.5% were male, 92.0% were delivered in hospital, 40.1% were migrants, 49.0% lived in rural areas, and 58.0% were within a distance of 5 km to the immunization clinics. Besides this, 65.6% of the surveyed households had one sibling, and 25.1% of the households had a monthly income of >10,000 CNY. Around 64.0% of the surveyed mothers were under 30 years of age, 79.4% of the surveyed mothers had a senior middle school background or above, 76.2% had jobs, and 74.0% had at least four visits to an antenatal clinic (ANC) (Table 1).

### 3.2. Vaccination Coverage and the Scar Evaluation

Table 2 shows that the coverage of BCG was 92.0% (95% CI: 90.6–93.5%). The coverage of PV3, DPT3, Hep B3, JEV1, and MR was 91.3% (95% CI: 89.8–92.8%), 90.5% (95% CI: 88.9–92.2%), 93.5% (95% CI: 92.2–94.8%), 91.1% (95% CI: 89.3–92.9%), and 90.7% (95% CI: 89.0–92.4%), respectively. From the 1282 children observed for the presence of a BCG-compatible scar, all of them had immunization cards and BCG vaccination records and 1245 children (97.1%) had developed the scars.

The multivariable logistic regression analyses revealed that the child born in hospital, higher maternal education, mother with no job, and resident child predictors were positively associated with a higher likelihood of being vaccinated with BCG (Table 3).

### 3.3. Timeliness of BCG

**Figure 1:**
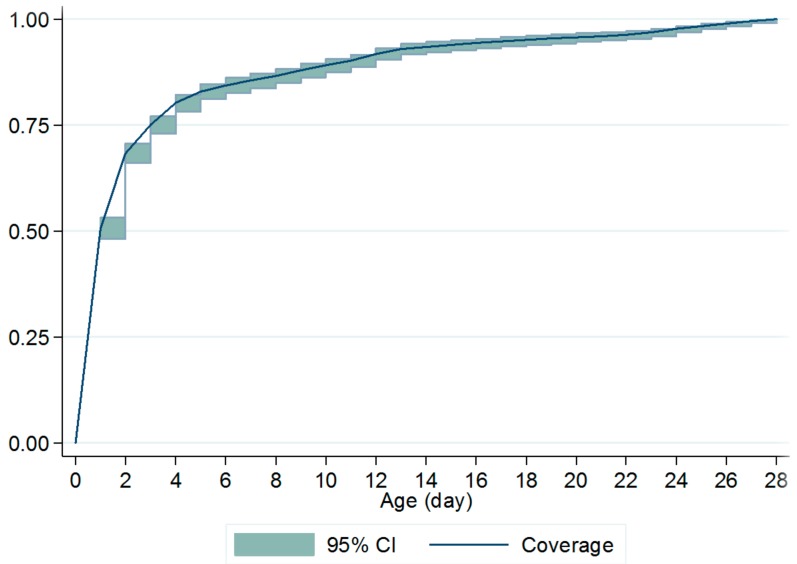
Inverse Kaplan–Meier curves showing the proportion of children immunized with BCG among the surveyed children with immunization cards (N = 1393).

Figure 1 represents the distribution of BCG vaccination administrated to children from the day of birth to 28 days old. The result indicated that 88.3% (1230) of the surveyed children received a BCG vaccination within the first 28 days of life.

The multivariable logistic regression analyses revealed that the less siblings in a family, child born in hospital, higher maternal education, mother with no job, and resident child predictors were positively associated with a higher timeliness of BCG vaccination (Table 4).

## 4. Discussion

This study provided a population estimate of BCG vaccination by using the most recent vaccination coverage survey data. It showed that the coverage of BCG was very high in children 24–35 months old, surpassing the coverage goal set by the Chinese center for disease control and prevention (CDC). However, the coverage of BCG in this study was a little lower than that derived from the Zhejiang provincial immunization information system (ZJIIS), which was 98.7% in children 24 months old [22]. It is known that the estimate from the ZJIIS was based on children who had already registered in the ZJIIS and children not registered in the ZJIIS would be more likely to be missed by routine immunization. Thus, the vaccination coverage derived from the ZJIIS might be overestimated when compared with the coverage from the field survey.

In this study, we found that the coverage of vaccine doses scheduled before the first birthday was over 90%. We thought that the enhancement of routine immunization program strategies (such as sending text messages as reminders to have administered all vaccines due) that had been conducted in every community since 2011 could potentially affect the vaccination-seeking behavior of children’s caregivers. However, there might be a selection bias since 13 children failed to present immunization cards. Although most of them had received immunization cards previously, if those who did not present the card had lower coverage, our estimates might represent a slight overestimation of the true coverage. Compared with other vaccinations scheduled before 12 months of age, the coverage of BCG was higher than that of the other vaccine doses studied except for HBV3. Administration of Hep B at birth is required for any registered maternity hospital in China, while the administration of BCG at birth is recommended but not mandatory [23]. As such, children born in maternity hospitals would be more likely to start a Hep B series or obtain a BCG vaccination and the coverage of these two vaccines would be higher than that of other vaccines. On the other hand, the coverage of BCG might be lower than that for Hep B as some children who did not receive the BCG vaccine in a timely manner would be exempted from BCG vaccination as their PPD readings were positive or they were over 4 years of age.

According to the immunization cards observed, very few children vaccinated with BCG failed to develop the scar. Our findings were consistent with similar studies in other countries, ranging from 1% to 20% [24,25,26,27]. Observer bias could have occurred as field investigators were not blind to the records from immunization cards. Recent studies showing beneficial effects associated with scars, such as a decreased mortality in children with scars, has opened an argument on re-vaccinating children who failed to develop a scar after the first BCG vaccination [3,6]. Some have also suggested that the BCG scar should be an indicator to monitor the performance of the immunization program [25].

The timing of vaccination is critical for obtaining timely protection, but also for being an indicator of non-adherence. We considered a BCG vaccination to be timely if it occurred within the first 28 days of life, which is the criterion recommended by the WHO. In this study, the proportion of delayed BCG vaccination was 11.7%, which was lower than the 33% found in another report from Tanzania [12]. However, definitions of delayed BCG vaccination differ from place to place. For example, some African countries considered it to occur after eight weeks after birth, thus comparisons with other studies should be made cautiously [11,13].

In this study, we found four determinants associated with BCG vaccination and its timely administration. First, as we mentioned before, a proportion of maternity hospitals provided a BCG vaccination together with the first dose of Hep B, which is mandatory for all maternity hospitals to give every newborn after birth. As such, children delivered at hospitals might have an increased likelihood to be vaccinated in a timely manner. Moreover, mothers who gave birth at hospital might be closer to the public health service and would have a better utilization of an immunization service [18]. Second, a higher maternal education level could assist mothers to communicate with health workers efficiently and have a positive impact on vaccination through a better understanding and acceptance of immunization knowledge. Previous reports have shown that a child whose mother has a lower education level is less likely to receive full immunizations [28,29]. Besides this, a mother with a higher education background would have a better awareness and capacity to take advantage of an immunization service [30,31,32,33]. Third, we assumed that mothers with jobs had less time to spare for childhood immunization as it might not be one of the priorities amidst other competing events to bring healthy children to obtain vaccinations. At the same time, previous studies have demonstrated that those mothers were less aware of the information on vaccination [34,35,36]. Fourth, we found that the patterns of utilization of a vaccination service of migrant children were not similar to those of resident children. Although the exact reasons need to be explored in future research, we attributed a missed or delayed BCG vaccination to the vulnerability of migrant people in a new sociocultural environment, the demand for the service, negative immunization experiences, and the capacity of providers [37,38,39]. Furthermore, there was another determinant only associated with the timeliness of BCG vaccination. Children with more siblings were found to have an increased probability to receive a BCG vaccination in an untimely manner. We assumed that families incurred a greater cost and required more resources to support more children, which might adversely affect health service utilization. Another study suggested that parental attention would be diverted by other children and the motivation for parents to prioritize immunization amidst competing demands for time was limited since the benefits of vaccination sometimes might not be immediately apparent [40].

This study had several limitations. First, selection bias could occur in evaluating the BCG coverage through scar reading since only children who were present at the moment of the interview were included. Secondly, although most of the children who could not present an immunization card stated that they had received one previously, these children might live in families with more difficulties in accessing the health system or not be able to have a proper follow up of their vaccination status; thus, our vaccination coverage could be overestimating the real one. Last, as this study focused only on one birth cohort, the results might be subjected to a cohort effect/bias if there was unusually good coverage in that cohort due to some unknown or uncertain reasons. We would like to continuously monitor the BCG coverage trends for a long period of time and explore its risk factors.

## 5. Conclusions

This study showed an optimal vaccination coverage of BCG. The vast majority of BCG vaccines were given within the first 28 days after birth. Scar development occurred in almost all infants. Several risk factors were identified for the lack of BCG vaccination or delayed BCG administration. Tailored interventions should be more greatly focused on and targeted to children not delivered at hospital, children whose mothers are less educated or have a fixed job, migrant children, and children with more siblings.

## Figures and Tables

**Table 1:** Summary distribution of the demographic characteristics of surveyed children with immunization cards (N = 1393).

Variables	*n*	%	Variables	*n*	%
Sex			Residence		
Male	718	51.5	Urban	710	51.0
Female	675	48.5	Rural	683	49.0
Number of siblings			Immigration status		
1	914	65.6	Resident	835	59.9
2	363	26.1	Migrant	558	40.1
≥3	116	8.3	Antenatal clinic (ANC) visits		
Place of delivery			none	55	3.9
Hospital	1281	92.0	1–3	307	22.0
Home	112	8.0	≥4	1031	74.0
Age of mother (years)			Distance to immunization clinic		
<30	892	64.0	<5 km	585	42.0
≥30	501	36.0	≥5 km	808	58.0
Maternal education level			Monthly household income per capita		
<senior middle school	287	20.6	<5000 CNY	272	19.5
≥senior middle school	1106	79.4	5000–10,000 CNY	772	55.4
Maternal employment status			>10,000 CNY	349	25.1
Home fulltime	331	23.8			
Employed	1062	76.2			

**Table 2:** Vaccination coverage among the surveyed children with immunization cards (N = 1393).

Vaccination Dose	Number of Children Immunized	%	95% CI
BCG	1282	92.0	90.6–93.5
PV3	1272	91.3	89.8–92.8
DPT3	1261	90.5	88.9–92.2
Hep B3	1303	93.5	92.2–94.8
JEV1	1269	91.1	89.3–92.9
MR	1263	90.7	89.0–92.4

BCG, Bacille Calmette-Guérin; PV, poliovirus vaccine; DPT, diphtheria-pertussis-tetanus vaccine; Hep B, hepatitis B; JEV, Japanese encephalitis virus live attenuated vaccine; MR, measles–rubella combined vaccine; CI, confidence interval.

**Table 3:** Univariate and multivariate analyses of the predictors associated with the BCG vaccination.

Variable	Predictors of the BCG Coverage			
	COR (95% CI)	*p*	AOR (95% CI)	*p*
Sex				
Male	1.0	-		
Female	1.0 (0.8–1.3)	0.95		
Number of siblings				
1	1.0	-	1.0	-
2	0.9 (0.7–1.6)	-	0.9(0.7–1.6)	-
≥3	0.6 (0.4–0.9)	<0.01	0.8(0.6–1.3)	0.07
Place of delivery				
Home	1.0	-	1.0	-
Hospital	2.5 (1.8–4.2)	<0.01	1.9 (1.4–3.0)	<0.01
Age of mother (years)				
<30	1.0	-	1.0	-
≥30	0.6 (0.3–0.8)	0.03	0.9 (0.7–1.2)	0.11
Maternal education level				
<senior middle school	1.0	-	1.0	-
≥senior middle school	2.5 (1.8–3.9)	<0.01	2.0 (1.4–2.7)	<0.01
Maternal employment status				
Home fulltime	1.0	-	1.0	-
Employed	0.7 (0.5–0.8)	0.03	0.8 (0.7–0.9)	0.04
Residence				
Urban	1.0	-	1.0	-
Rural	0.8(0.7–0.9)	0.02	0.9 (0.7–1.5)	0.18
Immigration status				
Resident	1.0	-	1.0	-
Migrant	0.6 (0.2–0.8)	<0.01	0.8 (0.6–0.9)	0.01
Antenatal clinic (ANC) visits				
none	1.0	-		
1–3	1.1 (0.8–1.3)	-		
≥4	1.3 (0.8–1.8)	0.15		
Distance to immunization clinic				
<5 km	1.0	-		
≥5 km	0.9 (0.8–1.6)	0.24		
Monthly household income per capita				
<5000 CNY	1.0	-		
5000–10,000 CNY	0.9 (0.8–1.5)	-		
>10,000 CNY	1.1 (0.9–1.8)	0.49		

Note: COR: crude odds ratio (OR) obtained from *χ^2^* test; AOR: Adjusted OR obtained from multivariate logistic regression analysis.

**Table 4:** Univariate and multivariate analyses of the predictors associated with the timeliness of BCG vaccination

Variable	Predictors of the Timeliness of BCG			
	COR (95% CI)	*p*	AOR (95% CI)	*p*
Sex				
Male	1.0			
Female	1.1 (0.8–1.3)	0.71		
Number of siblings				
1	1.0	-	1.0	-
2	0.8 (0.6–0.9)	-	0.9 (0.7–1.5)	-
≥3	0.6 (0.3–0.8)	<0.01	0.7 (0.5–0.9)	0.01
Place of delivery				
Home	1.0	-	1.0	-
Hospital	3.0 (1.9–4.6)	<0.01	1.9 (1.3–2.7)	0.03
Age of mother (years)				
<30	1.0	-	1.0	-
≥30	0.8 (0.6–0.9)	0.03	1.0 (0.9–1.1)	0.20
Maternal education level				
<senior middle school	1.0	-	1.0	-
≥senior middle school	4.1 (2.7–7.0)	<0.01	2.8 (1.9–4.5)	<0.01
Maternal employment status				
Home fulltime	1.0	-	1.0	-
Employed	0.7 (0.5–0.8)	<0.01	0.8 (0.6–0.9)	0.04
Residence				
Urban	1.0	-		
Rural	1.1 (0.9–1.3)	0.27		
Immigration status				
Resident	1.0	-	1.0	-
Migrant	0.5 (0.3–0.7)	<0.01	0.7 (0.4–0.8)	<0.01
Antenatal clinic (ANC) visits				
none	1.0	-		
1–3	1.0 (0.8–1.4)	-		
≥4	1.4 (0.8–2.1)	0.11		
Distance to immunization clinic				
<5 km	1.0	-	1.0	-
≥5 km	0.7 (0.5–0.8)	0.03	0.9 (0.8–1.7)	0.31
Monthly household income per capita				
<5000 CNY	1.0	-		
5000–10,000 CNY	1.1 (0.8–1.9)	-		
>10,000 CNY	1.3 (0.9–2.0)	0.19		

Note: COR: crude OR obtained from *χ^2^* test; AOR: Adjusted OR obtained from multivariate logistic regression analysis.
